# Thermo-Mechanical Characterization of Friction Stir Spot Welded AA7050 Sheets by Means of Experimental and FEM Analyses

**DOI:** 10.3390/ma9080689

**Published:** 2016-08-11

**Authors:** Gianluca D’Urso, Claudio Giardini

**Affiliations:** Department of Management, Information and Production Engineering—DIGIP, University of Bergamo, Via Pasubio 7b, Dalmine (BG) 24044, Italy; claudio.giardini@unibg.it

**Keywords:** FSSW, AA7050, process parameters, temperature distribution, welding force, mechanical properties, FEM simulation

## Abstract

The present study was carried out to evaluate how the friction stir spot welding (FSSW) process parameters affect the temperature distribution in the welding region, the welding forces and the mechanical properties of the joints. The experimental study was performed by means of a CNC machine tool obtaining FSSW lap joints on AA7050 aluminum alloy plates. Three thermocouples were inserted into the samples to measure the temperatures at different distance from the joint axis during the whole FSSW process. Experiments was repeated varying the process parameters, namely rotational speed, axial feed rate and plunging depth. Axial welding forces were measured during the tests using a piezoelectric load cell, while the mechanical properties of the joints were evaluated by executing shear tests on the specimens. The correlation found between process parameters and joints properties, allowed to identify the best technological window. The data collected during the experiments were used to validate a simulation model of the FSSW process, too. The model was set up using a 2D approach for the simulation of a 3D problem, in order to guarantee a very simple and practical solution for achieving results in a very short time. A specific external routine for the calculation of the thermal energy due to friction acting between pin and sheet was developed. An index for the prediction of the joint mechanical properties using the FEM simulations was finally presented and validated.

## 1. Introduction

When joining difficult-to-be-welded materials (typically Al, Ti, and Mg alloys, but also advanced high-strength steel [1]), it is normal practice, nowadays, to refer to friction stir welding (FSW) technology (patented by The Welding Institute in 1991 [2,3]). In particular, this technology has recently received large consideration from automotive and transportation [4,5], aeronautical [6], body-in-white, and other industries. This joining technique allows joining either similar or different materials [7,8,9,10,11], normally sheets, in butt, overlapping, and T-joint configurations [12,13].

The peculiarity of this technique is that the welding takes place by maintaining the solid state of the material. In fact, the rotational movement of the pin along the joining direction increases the material temperature for the combined effect of both friction between the tool and workpiece and internal friction within the stirred material. Therefore, the material is strongly deformed and stirred, generating highly resistant welds without melting. The combined effect of the plasticized material and the pressure applied by the tool shoulder causes the formation of a solid bonded region [14].

The FSW process can be used for joining thermoplastic and composite materials, as reported in [15,16,17,18,19,20], even if the main field of this application remains in joining high-strength materials [21,22,23,24,25].

Many variations of the pin geometry have been developed during the years for improving the stirring effect and for being suitable for different materials: with or without flutes [26,27], with conical or cylindrical shape [28], with either flat or no-flat shoulder shape [29], with refilling of the pin [30].

Different process configurations have been developed, too. Amongst them, friction stir spot welding (FSSW) has demonstrated to be effective for obtaining spot lap joints.

The FSSW technique, developed by Mazda Motor Corporation [31] and Kawasaki Heavy Industry [32], consists of a process in which the rotating pin moves into the overlapped sheets for a determined depth, stops for a while, and then is retracted: no transversal movement is present. The process parameters defining the joint mechanical characteristics [26] are the rotational speed of the pin, the plunge velocity, the dwell time, the maximum pin depth, and the pin and shoulder geometries (pin diameter, shoulder diameter, pin length). All of these parameters influence the heat generated, the process duration (that influences the temperature level and its distribution within the material), and the stirring effect. The main disadvantage of this technique is the keyhole remaining at the center of the welded volume that can be reduced using a retractable pin.

The heavy stirring of the material at the sheets’ interface as the pin penetrates in the lowers sheet, gives rise to a curling effect known as the hooking effect [33], which greatly influences the joint resistance of friction stir spot welds [34].

The large plastic flow and the heat generated determine remarkable microstructural modifications resulting in local changes of the material’s mechanical characteristics. In particular, starting from the base material (BM), without metallurgical modifications, and moving towards the pin axis, a heat affected zone (HAZ), where the material undergoes to temperature increase that modifies microstructure and mechanical properties, can be found. Even closer to the axis, the thermo-mechanically affected zone (TMAZ), where the material is heavily plastically deformed by the tool stirring action, can be observed. Finally, in the nugget, located in the middle of the joint, there is a recrystallized area where fine grains of uniform size replace the original grains [35,36]. For this reason, it is very important to understand the effects of process parameters and the setup on the weld quality and strength in terms of material microstructure, material resistance, and fatigue resistance.

In [37,38] the higher weld strength is related to the larger stirred area obtained, reducing the pin rotational speed. Other authors found that there is an optimal rotational speed range of the pin and that too low and too high speeds correspond to a low quality of the joint [39].

Other research focused on tool plunge rate/pin rotational speed ratio [40] rather than on the dwell time [41], demonstrating that these parameters can be optimized for obtaining sound parts.

Sakano et al. and Arul et al. [31,39] demonstrated that the shear load of lap joints first increases, and then decreases, for increasing values of the tool rotational speed.

Additionally, Freeney et al. and Tozaki et al. in [37,38] observed that the weld strength is related to the stirred zone size and they found that a reduction of the tool rotational speed can lead to an increase in weld resistance. In [40] the shearing resistance of the weld increases, while decreasing the tool rotational speed and increasing the tool plunge rate. On the contrary, other authors [42] found that the resistance of the joints decreases when decreasing the tool rotational speed.

It is evident that the effects of the process parameters on the joint properties are dissimilar among the various authors and, consequently, their influence on the joint characteristics is not yet fully understood. These discrepancies depend on the characteristics of joined materials, so it is important, from the practical point of view, to deepen the knowledge of the behavior of the particular alloy considered in order to identify the most suitable technological window.

In order to predict the joint behavior many researchers have developed analytical [43] and FEM models [44,45] of the process or focused their attention on the realization of artificial neural networks [46]. It must be stressed that to simulate the friction stir welding process represents a difficult task because of the high strain and strain rate occurring during the process involving non-linear material behavior, excessive mesh distortion, and very high computational efforts.

In [47,48] the temperature distribution and the plastic deformation were analyzed by using the ABAQUS code, considering an elastic–plastic deformation model. In [49] a 3D rigid-plastic model was set up in the DEFORM environment for studying welding force and temperature distribution as a function of the process conditions. In [50] an adaptive meshing technique has been introduced to preserve mesh quality under high strain conditions, while studying temperature, stress, and temperature-deformation aspects for aluminum alloy AA6061-T6 workpieces. In [51] the thermo-mechanical effects were analyzed in FEM environment considering a rigid tool and a deformable workpiece meshed using coupled temperature-displacement elements.

A valid alternative method for simulating the thermo-mechanical aspects occurring during FSSW can be based on FE codes supported by analytical models for predicting the heat generation at the pin-sheet interface [52]. In addition, FSSW cannot be simulated at steady-state conditions (as normally happen for FSW) because of its short cycle time (a few seconds). This means that the model validation must be based on experimental measurements of temperature and welding forces during the whole process.

In the present paper, an experimental study aimed to evaluate how the FSSW process parameters affect the temperature distribution in the welding region, the welding forces and the mechanical properties of the joints when considering AA7050 alloy, is reported.

The experimental data were also used for validating a simulation model of the FSSW process setup using the commercial code Deform 2D. An index for the prediction of the joint mechanical properties using the FEM simulations was finally presented and validated.

## 2. Experimental Procedure

The experimental campaign for studying the effects of welding process parameters on the thermal distribution in the welding region, on the welding forces and on the mechanical properties of FSSW lap joints, were performed on AA7050 aluminum alloy sheets having a thickness equal to 2 mm.

The sheets of 100 × 2 × 30 mm^3^ (L × T × W), were overlapped for 40 mm and welded on a CNC machine tool (EMCO Famup, Hallein, GmbH) using a cylindrical tool made of AISI 1040 steel with flat shoulder (pin length 3.5 mm, pin diameter 4 mm, shoulder diameter 12 mm). The chemical composition for the AA7050 alloy is reported in Table 1. Its average strength resistance was found equal to 498 MPa.

**Figure 1:**
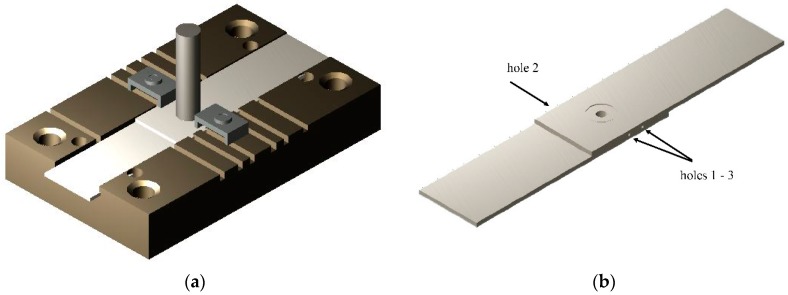
Experimental setup, (**a**) clamping system; and (**b**) details of a specimen with holes for the thermal analysis.

For measuring the temperature distribution during the welding process, three holes (with a depth equal to half of the specimen width and placed at 3, 4, and 7 mm from the specimen center) were realized. In this way three thermocouples (T1, T2, T3) were inserted at the sheets’ interface during the FSSW experiments. A specific clamping system was fabricated to block the specimens and the thermocouples (see Figure 1).

A Kistler piezoelectric load cell (Kistler Italia, Milano, Italy) was used to measure the welding forces in axial (Z) direction (Z load range = 0–10 kN, amplifier sensitivity of the used range = 0.5 mV/N, threshold < 0.01 N, sampling frequency = 30 Hz).

The tests were carried out varying tool rotational speed (S) (rpm), feed rate (F) (mm/min) (plunging speed) and plunging depth (d) (mm) according to Table 2, using a DOE approach (Factors: 3–Levels: 2–Base Design: 3; 8–Runs: 18–Replicates: 2–Blocks: 1–Center pts (total): 1, center point: S 3000–F 20–d 3.7). The dwell time (t) was 1 s for all the tests.

Shear tests were used for evaluating the mechanical properties of the joints using a universal testing machine with a 50 kN load cell, based on the UNI EN ISO 14273:2002 standard [53]. A traverse rate of 5 mm/min and a preload equal to 100 N were set. The mechanical resistance of the joints was investigated along a direction orthogonal with respect to the overlapping line. Two shim plates, having a thickness equal to 2 mm, were fixed on the edges of the specimens to avoid possible bending moments. 

## 3. Analysis of the Results

### 3.1. Welding Forces and Temperature

The measured welding forces and temperatures were analyzed by means of analysis of variance (ANOVA). A good repeatability with low data scatter was observed for all of the cases and a dependence of both welding force and temperature from rotational speed (S), feed rate (F), and plunging depth (d) was observed.

**Figure 2:**
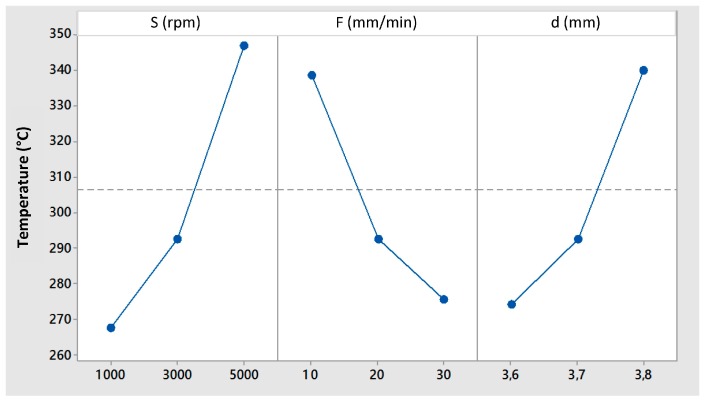
Main effects plot for temperature (T3).

Figure 2 shows the main effects plot for the maximum temperatures measured by the thermocouple T3 (7 mm from the joint axis). A significant increase of temperature for increasing values of rotational speed can be observed. A similar effect can be observed for increasing values of the plunging depth, while an opposite effect is related to the increase of feed rate. In effect, the tool rotation speed can be directly related to the power transferred to the workpiece. A similar consideration can be done for the plunging depth: an increase of this parameter generates an increase in the axial pressure and, consequently, in the heat generated by the friction. The opposite effect of the feed rate on the temperature can be easily explained, referring to the time for which the tool stays in contact with the parts that is inversely proportional to the tool feed. This means that the higher the feed, the lower the time in which the tool generates heat. Consequently, the maximum reached temperature reduces. No interaction between the process parameters has been identified.

**Figure 3:**
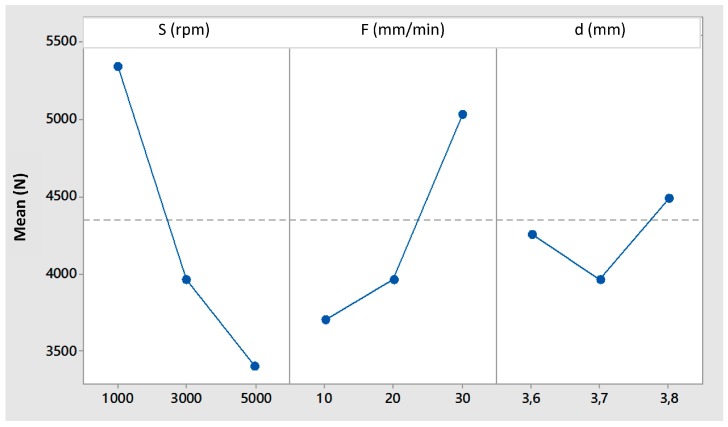
Main effects plot for welding forces.

Figure 3 shows the main effects plot for the maximum welding forces measured during the FSSW tests. It is evident how an increase of tool rotational speed, causing an increase in the sheet temperature, results in a reduction of the axial welding forces. An opposite effect is given by the feed rate. The plunging depth increase gives rise to an initial reduction of forces, but this trend is inverted for higher penetration values, when probably the reduction of the force due to the material softening is exceeded by the high deformation imposed to the material by the pin shoulder.

**Figure 4:**
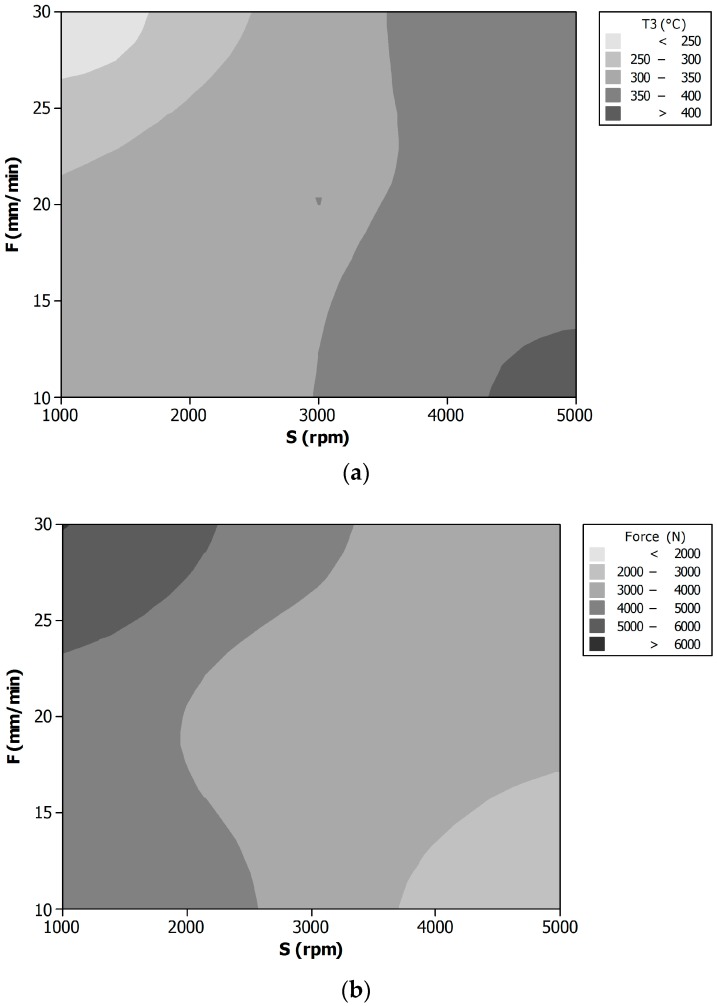
Contour plot of temperature T3 (7 mm from the joint axis) (**a**) and welding force (**b**) vs. tool rotational speed (S) and feed rate (F).

A more clear and complete relation among process parameters, temperature, and welding force is given by Figure 4, where the contour plot of temperature T3 (at 7 mm from the joint axis) (a) and of welding force (b) vs. tool rotational speed (S) and feed rate (F) are shown. These contour plots represent the iso-level curves for the response surfaces, i.e., the interpolating surface of the experimental observations for the different (S) and (F) values. In this way, it is possible to see the combined effect of the two process parameters in terms of T3 maximum temperature and welding force.

### 3.2. Shear Test

**Figure 5:**
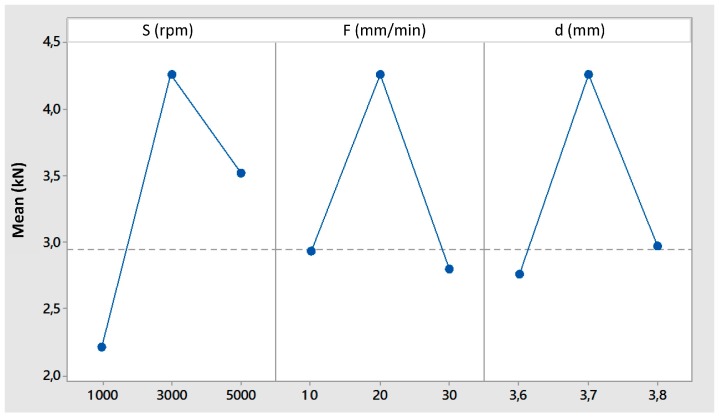
Main effects plot for maximum shear strength.

**Figure 6:**
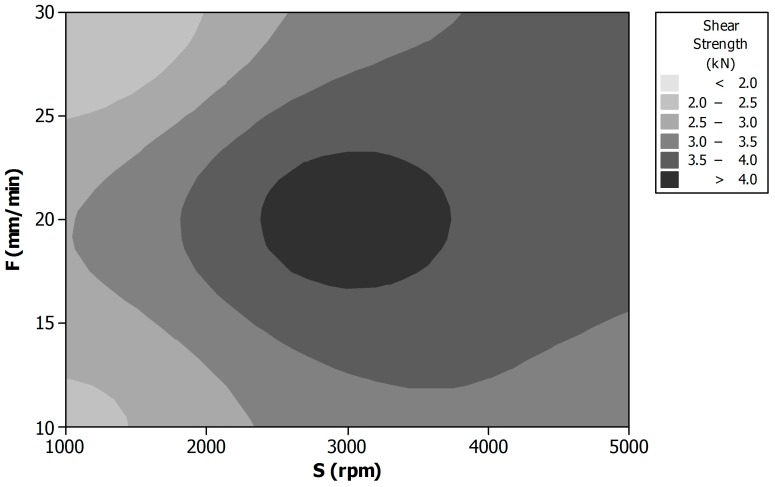
Contour plot of shear strength vs. tool rotational speed (S) and feed rate (F).

Figure 5 shows the main effects plot for the maximum shear strength. It is possible to observe how the maximum joint resistance is obtained for the intermediate values of all the process parameters. This is due to the compromise between the material stirring and heating effects that can be reached for the intermediate values of (S) and (F). Differently from temperature and welding force, in this case a certain interaction between the process parameters was identified. The complete combined effect of speed (S) and feed rate (F) on the shear strength is shown in the contour plot reported in Figure 6. It is evident how the maximum shear resistance (above 4 kN) is reached for the intermediate values of the two process parameters (the darkest area of the plot).

This plot is fundamental in identifying the process window for which the maximum shear strength can be expected.

From the practical point of view, it is convenient, once the optimal value of (d) is identified, to keep it as constant as possible since small variations of this parameter greatly influences the shear resistance of the joints. On the other hand, the other two parameters play more or less the same influence. This is why the technological window is given for (S) and (F). Small percentage variations of these parameters do not heavily influence the joint resistance.

**Figure 7:**
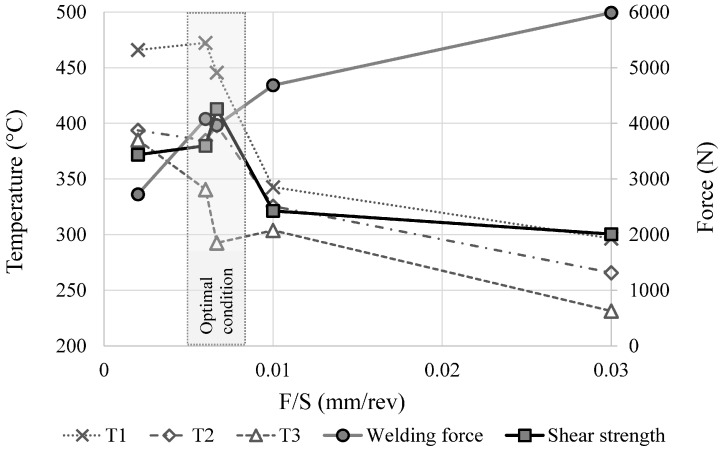
Welding force, shear strength, and maximum temperature (in T1, T2, and T3) as a function of the feed rate per unit revolution F/S (mm/rev).

In order to give a more concise representation of the results, Figure 7 shows welding force, shear strength, and maximum temperature (in T1, T2, and T3) as a function of the feed rate–rotational speed ratio (F/S) representing the feed rate per revolution (mm/rev). This parameter is strictly related to the thermal contribution and can be considered a valid index for the simultaneous representation of speed and feed effects.

An optimal condition in terms of strength can be observed for low values of F/S (the gray area in Figure 7 corresponding to intermediate values of welding forces and high values of temperature.

## 4. Simulation of the FSSW Process

### 4.1. FEM Model

To easily and quickly simulate a FSSW operation in terms of generated heat, temperature distribution, and force required, a 2D FEM model has been developed considering that the actual process can be seen as axisymmetric, even if in such a model it is not possible to simulate the pin rotation and, consequently, the heat generated. For this reason, a specific simulation approach has been adopted for computing the pin heat generation [52].

**Figure 8:**
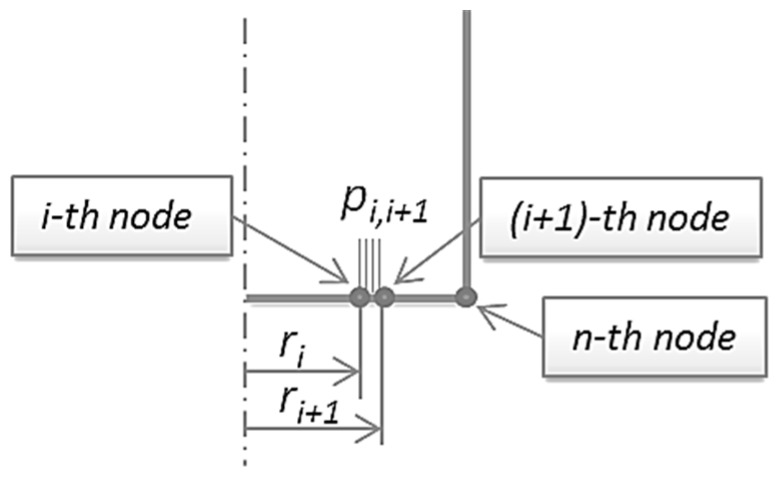
Scheme of the 2D FEM model of the pin.

The proposed approach is based on the development of an analytical model associated to the 2D FEM solver able to calculate the heat flux. A schematic description of the analytical model is reported in Figure 8 and presented in the following.

In particular, the heat flux is due to the mechanical power loss for friction acting between the pin and the sheet per unit area.

This power is the sum of the various contributions that can be calculated for each element of the pin in contact with the sheet (see Figure 8). For each boundary element, the FEM solver is able to calculate the pressure (p_i,i+1_) acting between the pin and the part. These pressure values, different from element to element, are saved at the end of each simulation step in the FEM database file. Afterwards, the FEM solver is stopped and the control is passed to the external program that analytically calculates the friction forces acting for each element (generically defined by the i-th and the (i + 1)-th boundary nodes of the pin) multiplying the pressure by the friction coefficient (µ) and by the contact area of the single element, i.e.: $2\cdot \pi \cdot \left(r_{i}+r_{i+1}\right)/2\cdot \left(r_{i+1}-r_{i}\right)\cdot p_{i,i+1}\cdot \mu$.

The power due to friction is then evaluated for each pin element as the product of the friction force and its average tangential velocity is equal to the mean radius of the element $\left(r_{i}+r_{i+1}\right)/2$ multiplied by the angular velocity (ω) of the pin.

This means that the heat generated for each element of the pin in contact with the sheet is:

(1) $$q_{i,i+1}=2\cdot \pi \cdot {\left(\frac{r_{i}+r_{i+1}}{2}\right)}^{2}\cdot \left(r_{i+1}-r_{i}\right)\cdot p_{i,i+1}\cdot \mu \cdot \omega$$

It is now possible to evaluate the heat flux (W/m^2^) by dividing the heat just calculated by the contact area of the single element, i.e.:

(2) $$\Phi \left(q_{i,i+1}\right)=\frac{q_{i,i+1}}{2\cdot \pi \cdot \left(\frac{r_{i}+r_{i+1}}{2}\right)\cdot \left(r_{i+1}-r_{i}\right)}=\frac{2\cdot \pi \cdot {\left(\frac{r_{i}+r_{i+1}}{2}\right)}^{2}\cdot \left(r_{i+1}-r_{i}\right)\cdot p_{i,i+1}\cdot \mu \cdot \omega }{2\cdot \pi \cdot \left(\frac{r_{i}+r_{i+1}}{2}\right)\cdot \left(r_{i+1}-r_{i}\right)}$$

(3) $$\Phi \left(q_{i,i+1}\right)=\left(\frac{r_{i}+r_{i+1}}{2}\right)\cdot p_{i,i+1}\cdot \mu \cdot \omega$$

The so-calculated heat flux does not take into account the internal friction of the material due to the stirring effect just below the rotating pin. For this reason, a k_c_ factor (equal or greater than 1) multiplying the heat flux values has been introduced. The k_c_ value was identified by means of an initial tuning phase of the model matching the experimental and FEM temperature distributions. The k_c_ value was set equal to 1.12.

At this point, it is possible to rewrite the FEM database, including boundary conditions at the pin-sheet interface the heat flux just calculated, and then the control returns to the FEM engine and one more simulation step is carried out. This procedure is repeated until the entire pin stroke has been simulated. The pin and the backplate are modeled as rigid bodies with heat transfer characteristics (H13 material), while the sheets are modeled as a unique part (4 mm thickness) with plastic elements. The flow stress of the material (AA7050 alloy) takes into account both work hardening and thermal effects. The mesh size of the sheets ranges between 0.16 mm far from the pin and 0.04 mm in the welding area. The FEM program used is Deform 2D plus the external program able to manage the thermal contribution deriving from the pin rotation as described above.

The choice of modelling the two sheets as a single body speeds up the calculus without introducing unacceptable simplification, being that the contact pressure between the sheets in the welding area very high.

**Figure 9:**
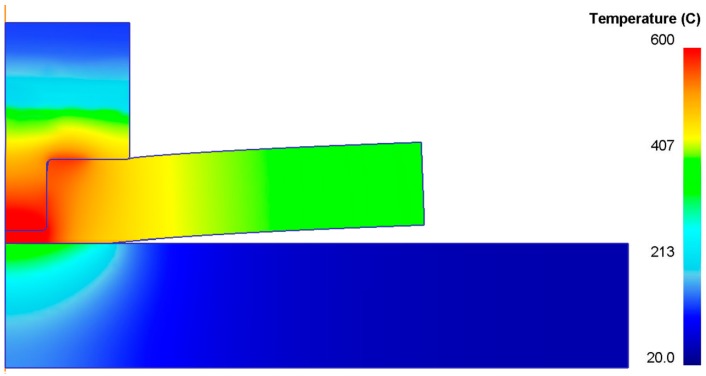
Example of FEM output (S = 5000 rpm, F = 10 mm/min, d = 3.8 mm).

Figure 9 shows an example of the FEM output in terms of temperature distribution (S = 5000 rpm, F = 10 mm/min, d = 3.8 mm).

### 4.2. Model Validation

**Figure 10:**
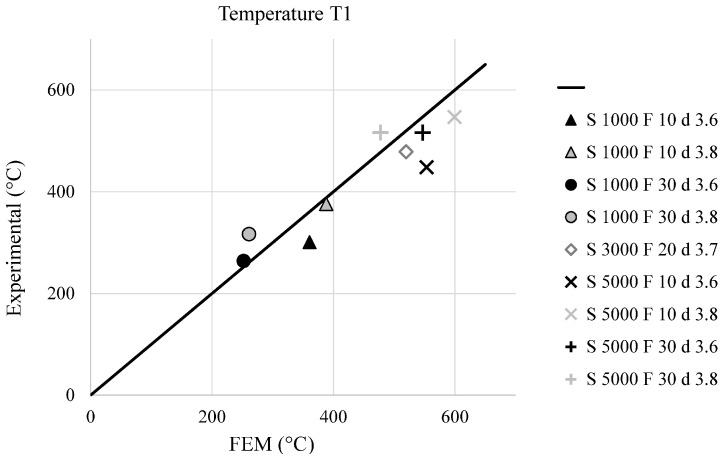
Comparison between experimental and simulated maximum temperatures at 3 mm from the joint axis, for the different welding conditions.

**Figure 11:**
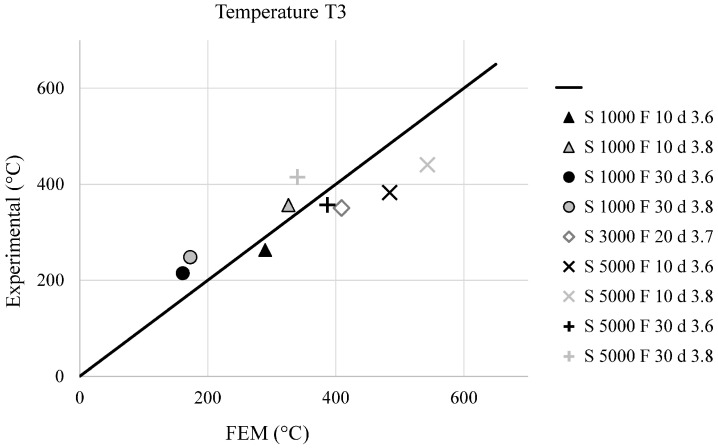
Comparison between experimental and simulated maximum temperatures at 7 mm from the joint axis, for the different welding conditions.

**Figure 12:**
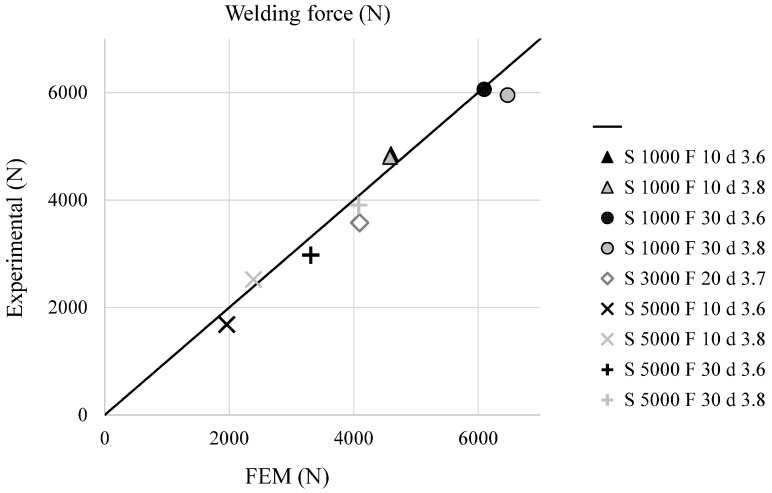
Comparison between experimental and simulated axial welding force for the different welding conditions.

The validation of the described FEM model was performed comparing numerical and experimental data. In particular, Figure 10 and Figure 11 show a comparison (experimental vs. FEM) in terms of temperature distribution for T1 and T3 (placed at 3 and 7 mm from the joint axis), while Figure 12 shows the comparison of the axial welding forces. It is evident how a satisfactory matching was achieved in terms of both maximum temperature and welding force prediction with an average estimation error ranging between 10% and 20%.

### 4.3. Predictive Index of the Joint Resistance

As the experimental tests have highlighted, there is a strict correlation between the process parameters and the joint resistance. A further study was, therefore, carried out trying to identify this correlation by using the FEM simulation output.

Referring to the results reported in the previous paragraphs and considering that the weld takes place at solid state, it is possible to state that both the pressure applied by the tool during the welding process (and consequently the mean or hydrostatic stress in the welding region) and the welding temperature, affect the joint quality in terms of shear strength.

In particular, the mean stress estimated by the FEM simulations in correspondence of the final tool displacement decreases for increasing values of rotational speed, and then for increasing values of the temperature in the joint region. Then, the mean stress increases for increasing values of the feed rate that means, also in this case, decreasing values of temperature.

Based on this considerations, a predictive index (*Ri*) of the joint resistance was defined taking into account the mean stress and the welding temperature as follows:

(4) $$Ri=\frac{\left|\sigma_{idr}\right|T^{2}}{k}$$

where σ*_idr_* is the mean stress, *T* the maximum temperature measured in the welding region, and *k* a constant value (related to the workpiece material). The *k* value for the considered case is equal to 7.8 × 10^6^.

**Figure 13:**
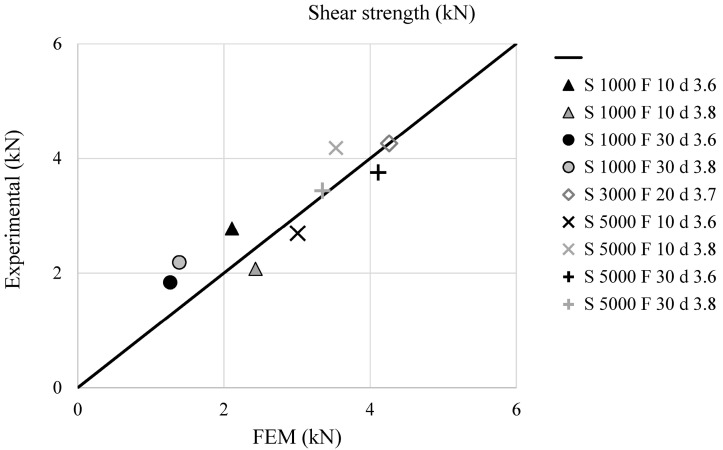
Comparison between shear strength (experimental) and resistance index (calculated from the FEM results) for the different welding conditions.

Figure 13 reports the comparison between experimental and simulation results in terms of shear strength and resistance index for the tested conditions, showing a good agreement for all of the cases.

## 5. Concluding Remarks

In the present paper, an experimental study on how the FSSW process parameters affect the thermal distribution in the welding region, the welding forces, and the mechanical properties of the joints for two sheets made of AA7050 alloy is presented. The dependence of welding forces and maximum achieved temperature on rotational speed and feed rate was studied, showing how the reduction of the F/S ratio leads to lower welding forces and higher welding temperatures. The mechanical properties of the joints are strongly related to these aspects, too. In particular, the shear resistance of the joints is the maximum for intermediate values of temperature and welding forces, that is, when the material temperature and pressure are high enough for the solid state phenomena occurrence. Moreover, an increase of the shear resistance can be observed for increasing values of plunging depth. The technological window able to guarantee the maximum shear resistance has been identified. From the practical point of view, the plunging depth should be kept constant at its intermediate value due to its high effect on the joint resistance.

A 2D very fast simulation model for the evaluation of thermo-mechanical effects in FSSW was also proposed and described in this study, showing its ability in representing the actual phenomena. The results of the simulations, once validated, allowed deriving important information used for the calculus of the proposed resistance index. The latter, based on mean stress and maximum temperature in the welding region, was finally validated showing its good ability in predicting the joint shear resistance. 

## Figures and Tables

**Table 1:** Chemical composition for the AA7050 alloy (min − max %).

Al	Cr	Cu	Fe	Mg	Mn	Si	Ti	Zn	Zr
87.3	≤0.04	2.0	≤0.15	1.9	≤0.10	≤0.12	≤0.06	5.7	0.08
90.3		2.6		2.6				6.7	0.15

**Table 2:** Welding conditions for the thermal and forces analyses.

Rotational Speed (S) (rpm)	Feed Rate (F) (mm/min)	Plunging Depth (d) (mm)
1000–5000	10–30	3.6–3.8
